# A Hybrid Strategy for Profile Measurement of Micro Gear Teeth

**DOI:** 10.3390/mi14091729

**Published:** 2023-09-02

**Authors:** Guangyao Huang, Jiao Bai, Feng Feng, Long Zeng, Pingfa Feng, Xinghui Li

**Affiliations:** 1 Shenzhen International Graduate School, Tsinghua University, Shenzhen 518055, China; huang-gy22@mails.tsinghua.edu.cn (G.H.); zenglong@sz.tsinghua.edu.cn (L.Z.); feng.pingfa@sz.tsinghua.edu.cn (P.F.); 2 Institute of Materials, China Academy of Engineering Physics, Mianyang 621900, China; baij_07@126.com; 3 Tsinghua-Berkeley Shenzhen Institute, Tsinghua University, Shenzhen 518055, China

**Keywords:** micro gear teeth, profile measurement, error separation, hybrid measurement, flexspline

## Abstract

A hybrid strategy is proposed to meet the challenge of obtaining the profile of micro gear teeth with a small modulus. Firstly, the contact probe segmentally obtained the falling flank profiles with an auxiliary lifting mechanism to avoid interference when it climbs on the rising slope. Then, the noncontact chromatic confocal displacement sensor efficiently acquired the gear peak positions to carry out the two-point error separation with the gear peak positions from the probe measurement. Finally, actual experiments were carried out to obtain the profile of a harmonic drive flexspline. Compared with the commercial ultraprecise profiler, the proposed method provides measurement results with a deviation of less than 20 μm. In conclusion, the hybrid strategy is feasible and accurate for drawing the micro gear teeth profile without any collision between the measuring probes and the measured workpiece.

## 1. Introduction

Micro gears are widely used in robotics, aerospace, and manufacturing fields as the essential components for the motion/force transition. They are usually manufactured on a linear or circular substrate. Harmonic gear transmission is a novel technology for circular substrates with many advantages including simple structure, large transmission ratio, stable motion, and low noise. One set of harmonic reducers comprises a wave generator, a flexspline, and a circular spline. The wave generator drives the flexspline to deform and then rotate the circular spline. The gear qualities of the flexspline and the circular spline greatly influence the power transmission efficiency. The design and manufacturing of the gear teeth require a precise profile to accurately evaluate the gear quality or wear status of the flexspline [1,2].

Harmonic gears are usually designed with small gear module and dozens of teeth, increasing the measuring difficulty. Moreover, the flank profile of the gear tooth is usually steep, and the shape of the gear teeth is often irregular (such as involute or non-involute) [3,4].

With the rapid development of harmonic drive technology, a variety of tooth profile measuring methods have been reported, including contact and noncontact methods: the three-coordinate method, stylus profiler methods, laser interferometer method, chromatic confocal method, structure light method, visual image method, and so [5,6,7,8,9,10,11]. Among these methods, the laser interferometer method, structure light method, and visual image method can only provide the maximum section profile with limited visual field, lateral resolution, and systematic imaging aberration.

The three-coordinate method and stylus profiler method are both contact modes. The probe contacts the tooth surface to indicate the position of the touchpoint, achieving the profile by scanning. Usually, the contact mode is stable and reliable with a wide range of applications. However, the probe may cause systematic errors at some uncertain touchpoints, and interference may occur during the scanning, damaging the probe or the tooth. Thus, it is necessary to apply a fine probe and define the scanning path in advance, and then perform error compensation with a calibrated algorithm. Moreover, the steep surface may easily decrease the pressure angle between the inclined plane and the probe, causing frictional self-locking, jamming, or even damage [12].

On the other hand, an optical probe can also provide radial positions of the gear surface to acquire the gear profile without direct contact with the tooth surface, which is efficient and safe. Optical probes may come from commercial instruments, such as laser triangulation sensors, chromatic confocal sensors, laser interferometry displacement sensors, and so on. These optical methods often need enough reflected light to extract position information. However, the steep surface only reflects a little light, making it difficult to acquire a complete tooth profile [3,13]. In addition, the laser interferometer method, structure light method, and visual image method can only provide the maximum section profile with limited visual field, lateral resolution, and systematic imaging aberration.

Except for the difficulty caused by the measuring probe, gear installation error, instrument error, and rotation error also affect the measuring accuracy [14,15]. Although the error separation technology has been widely used in cylindrical profile measurement, such as the two-point method, rotary method, multi-step method, and three-point method, there is a lack of relevant research on the application for gear tooth profile measurement [16,17,18,19,20,21,22].

Therefore, this paper proposed a hybrid measuring strategy to overcome the limitations of conventional methods for harmonic gear tooth profile measurement, with a contact metal probe and a non-contact chromatic confocal probe. The rotating platform drives the flexspline to perform circular scanning, while the metal and optical probes provide radial positions on the gear surface. The metal probe is supported with air to freely descend along the tooth surface, and a push–pull mechanism is used to assist the probe lifting during the climbing stage. At the same time, another set of tooth peak points is obtained using a chromatic confocal sensor. With the tooth peak points by the metal probe and the valley points by the chromatic confocal probe, the installation error and rotation error are corrected through the two-point error separation method. In all, the whole teeth profile is achieved by connecting the segmented gear profiles and the corrected peak points.

## 2. Measurement Principles

**Figure 1:**
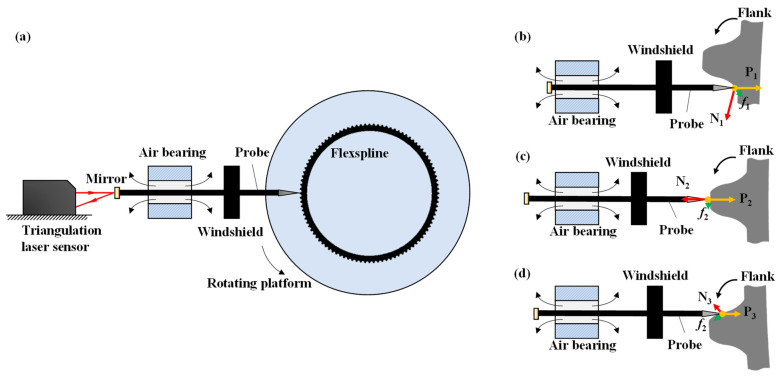
(**a**) Tooth profile measurement of flexspline by probe method; (**b**) force analysis at climbing stage; (**c**) force analysis at steady stage; (**d**) force analysis at descending stage.

As shown in Figure 1a, a flexspline profile measurement system is designed based on a rotating platform and a metal probe. The probe only moves left and right in the horizontal direction, and the flexspline is fixed on the rotating platform for rotational scanning. In addition, an air bearing is used to suspend the probe to drive it back and forth in the axial direction to provide the axial displacement with a laser displacement sensor. A windshield is fixed to the probe to provide a certain pressure (about 10 mN) on the flexspline to tightly maintain contact.

However, the probe may be easily deflected or even stuck in the measurement process because of the unbalance forces. Figure 1b–d show the climbing, steady, and descending stages of the measurement, respectively. As shown in Figure 1d, when the probe descends along the flank surface, the resultant force on the tip of the probe can reach a balanced state, so the entire probe will only move back and forth following the tooth surface. The movement of the stylus can thus inversely deduce the profile of the flexspline. As shown in Figure 1b, when the probe climbs along the flank, the stuck phenomenon occurs frequently. The analysis of the force on the probe shows that when the probe climbs along the flank, the flexspline surface provides a support force (N_1)_ to the probe. The resultant of the friction force (f_1_), the preload force (P_1),_ and the support force (N_1)_ faces the lower-right, making the probe easy to rotate. When the air bearing cannot balance the rotary moment, the probe will deflect to the opposite inner wall. Figure 1c shows a good balance between the probe and the flexspline to acquire accurate peak information. Briefly, although the descending side can generally be completed smoothly, the probe will be tilted to a certain extent or even stuck on the ascending flank due to the unbalanced moment, which will increase measurement error or even interrupt the measurement [2,23].

**Figure 2:**
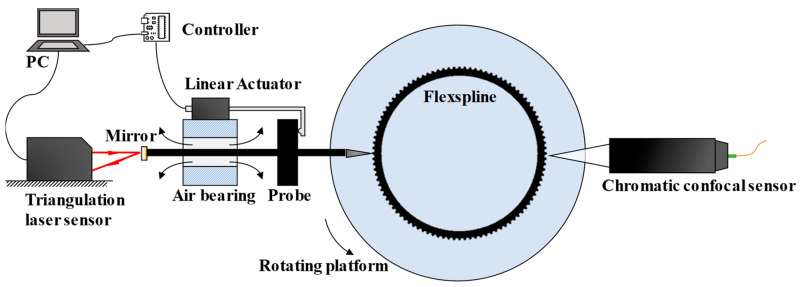
Profile measurement system with the proposed hybrid strategy.

In response to the results obtained by previous researchers, this paper proposed a solution by adding an auxiliary lifting device for the probe to skip the measurement of the climbing stage, as shown in Figure 2. In this way, both sides of the tooth profile can be obtained with forward and reverse rotations. The linear actuator moves forward or backward, depended on the real-time displacement data from the triangulation laser sensor. Simultaneously, a chromatic confocal sensor is set opposite the metal probe to provide peak displacement data.

**Figure 3:**
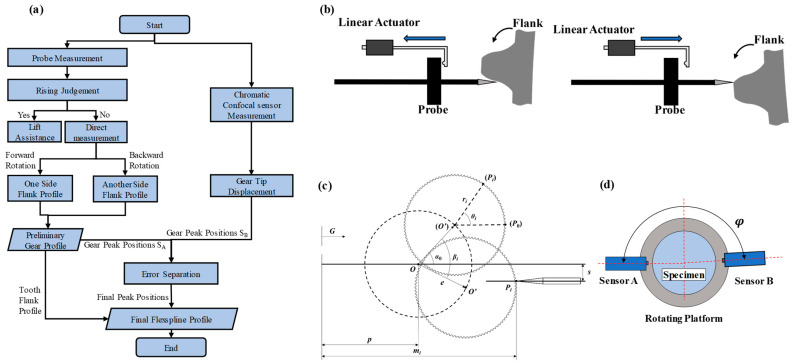
(**a**) Detail measuring procedure; (**b**) auxiliary lifting strategy; (**c**) installation error mathematic model; (**d**) two-point method.

The measurement procedure is shown in Figure 3a. The metal probe is used to acquire the tooth flank profile and the gear peak positions. Meanwhile, the chromatic confocal sensor is used to obtain the gear peak positions to carry out error separation with these gear peak positions from the probe measurement [24,25,26]. Then, the final flexspline profile can be acquired by connecting the tooth flank profile and the final gear peak positions. During the probe measurement, an auxiliary lifting strategy is employed to avoid collision, as shown in Figure 3b. The linear actuator drives the probe backward in its rising stage, and releases the probe in its falling stage. After one side of the flank profile is obtained, another side of the flank profile will be gained from the reverse rotation. The two flank profiles are combined as the preliminary gear profile. Lastly, the final flexspline profile is completed with the tooth flank profile and the final peak positions.

Figure 3c presents an installation error in the mathematical model of the probe measurement system, where *O* is the center of the rotating platform, *O’* is the center of the gear, *G* is the measurement zero position of the sensor, e is the installation error between the center of the gear and the center of the rotating platform, *s* is the alignment error between the axis of the probe and the center of the rotating platform, α_0_ is the eccentric angle of the gear at the initial position, p is the distance between the center of the rotating platform and the measurement zero position, and m_i_ is the measured value of the sensor. When the gear rotates around *O* for an angle β_i_, ∠PiO’P_0_ is the corresponding angle of the gear, namely θ_i_. Based on this model, the relationship between the measured value and the corresponding radius of the gear can be established, as shown in Formula (1), where r_i_ is the corresponding radius value of the gear.

(1) $$m_{i}=p+e\times \cos(\beta_{i}-\alpha_{0})+\sqrt{r_{i}{}^{2}-{\left[e\times \sin(\beta_{i}-\alpha_{0})-s\right]}^{2}},$$

Then, the radius value r_i_ of the gear at the angle of θ_i_ can be obtained in Formulas (2) and (3).

(2) $$r_{i}=\sqrt{\left(m_{i}-p-e\times \cos{\left(\beta_{i}-\alpha_{0}\right)}^{2}+{\left[e\times \sin(\beta_{i}-\alpha_{0})-s\right]}^{2}\right)},$$

(3) $$\theta_{i}=\beta_{i}+\arctan\frac{e\times \sin\left(\beta_{i}-\alpha_{i}\right)-s}{\sqrt{r_{i}{}^{2}-{\left[e\times \sin\left(\beta_{i}-\alpha_{i}\right)-s\right]}^{2}}},$$

Furthermore, the two-point method is used to separate the installation error (e) and the rotation error, as shown in Figure 3d. As the gear peak is measured at an ideal state to possess a high degree of confidence, only the gear peak data are used in the two-point method for accurate gear peak positions. Then, the probe and chromatic confocal sensor are approximately coaxial across the rotation center to obtain the gear peak positions of S_A_ and S_B_. The included angle between the probe and the chromatic confocal sensor is accurately set as φ. According to the mathematical assumption, the profile coordinate S can be expressed as follows.

(4) $$S=c_{0}S_{A}\left(\theta \right)+c_{1}S_{B}\left(\theta \right)=c_{0}r\left(\theta \right)+c_{1}r\left(\theta +\varphi \right),$$

Among them, $c_{0}=1$, $c_{1}=-1/\cos(\varphi )$. Then, the following formula can be obtained using Fourier transformation.

(5) $$S\left(\omega \right)=c_{0}R\left(\omega \right)+c_{1}R\left(\omega \right)e^{i\omega \varphi },$$

After extracting the common factor of this formula, R(ω) can be expressed by the following formula.

(6) $$R\left(\omega \right)=S\left(\omega \right)/H\left(\omega \right),$$

Among them, H(ω) is the weight function as follows.

(7) $$H\left(\omega \right)=c_{0}+c_{1}e^{i\omega \varphi },$$

Using inverse Fourier transformation of R(ω), the final gear peak positions can be accurately separated from the measured displacement of the probe and the chromatic confocal displacement sensor.

## 3. Experiment and Analysis

**Figure 4:**
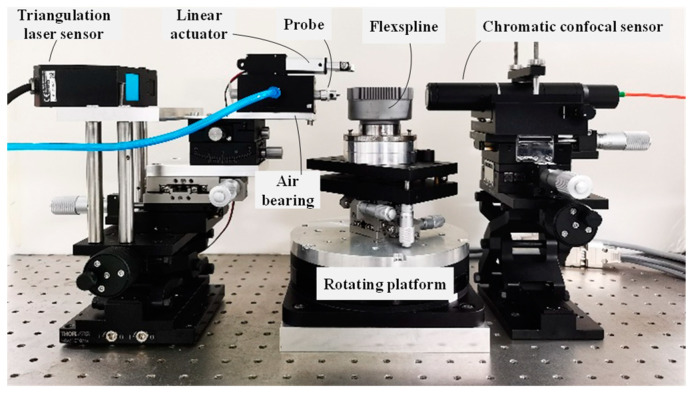
Flexspline tooth profile measurement system.

Figure 4 depicts the setup of the measurement system. GJCS-25-80 is used as the target flexspline with 160 teeth, 700 μm tooth height, and 1237 μm tooth pitch, which is produced by IPE Group Limited. The maximum flank slope is approximately 74°, and its outside diameter is 63 mm. It is fixed on a precision rotating platform (TD-170-50), whose rotating accuracy is 30”, and repeatability is 2.5”. A triangulation laser sensor (Keyence LK-G30) is used to detect the displacement of the self-made probe, with a measuring range of 25 mm and a repeatability of 0.05 μm. To minimize the morphological filtering effect and improve the measurement accuracy, the straightness and roundness of the probe are made to be less than 3 μm with a tip radius of about 2 μm. Furthermore, a chromatic confocal sensor is set opposite to the probe with an included angle of 179.56°.

**Figure 5:**
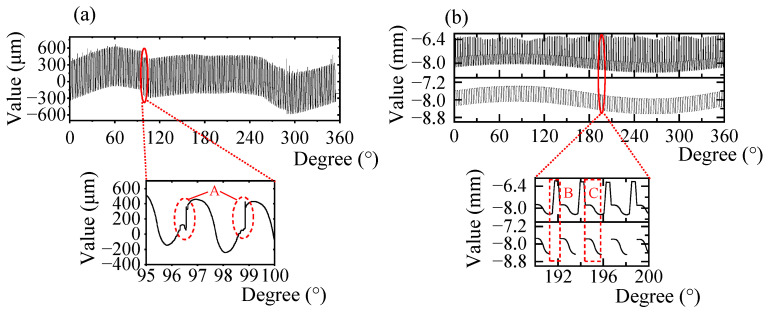
(**a**) Measurement results using the traditional probe method; (**b**) measuring results from the auxiliary lifting method.

First, the flexspline is measured without adding the auxiliary lifting device. In order to keep the scanning process as stable as possible, the rotating speed of the rotating platform was set as small as 0.2°/s. Since the target flexspline has numerous microstructures, the probe tip constantly experiences reciprocating swings, as shown in Figure 1. During the climbing stage, the probe deflects due to the unbalanced torque, making the actual measurement process bumpy, and obvious fluctuation of the curve can be observed in Figure 5a. The enlarged image of the tooth shape shows that the measurement results in the descending stage are relatively normal, whereas the results in the climbing stage are irregular, as shown in area A. This indicates that the stuck phenomenon considerably affects the measurement or damages the probe and the gear surface. Even if the rotating speed is reduced to 0.01°/s, the stuck phenomenon continues to occur. Hence, the auxiliary lifting device is designed and added to the measuring system. Figure 5b shows the measurement results.

In Figure 5b, the upper part shows the output displacement data of the triangulation laser sensor with periodic fluctuations caused by different teeth profiles. The enlarged view shows that many spikes exist in the rising stage when the probe is lifted. When the probe contacts the gear surface again, there will be a slight jitter. The contact point can be obtained by identifying the position of the jitter. The gear peak points can be obtained by the valley of the profile. Therefore, the descending profile can be obtained by removing the spikes and rising flanks. Since the probe starts to contact the tooth surface before contacting the tooth peak, the measurement error between the gear peak and the descending profile is negligible [27].

**Figure 6:**
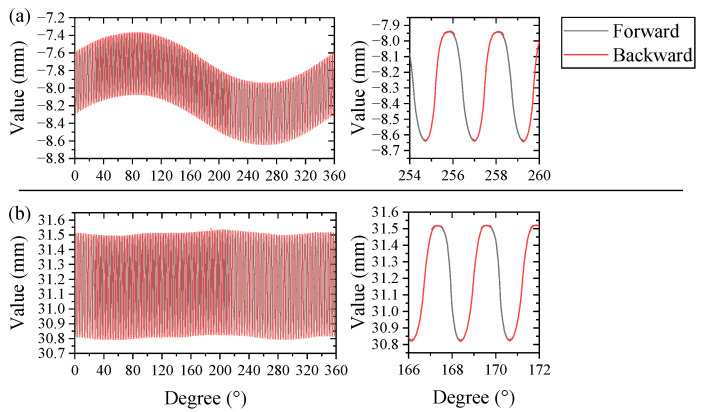
(**a**) Simply stitched flexspline profile; (**b**) synthetic profile after separating the eccentricity error and alignment error.

The whole gear profile can be obtained by directly connecting the segmented gear profiles from the forward rotation measurement and the backward rotation measurement, as shown in Figure 6a. However, the installation error caused serious fluctuation in the profile. Thus, Formulas (1)–(3) are used to separate the installation error, and the preliminary flexspine profile is shown in Figure 6b. In detail, the values of e, p, α_0,_ and s can be obtained from the curve fitting process by substituting the rotation angle and the laser sensor value into β_i_ and m_i,_ as shown in Table 1.

**Figure 7:**
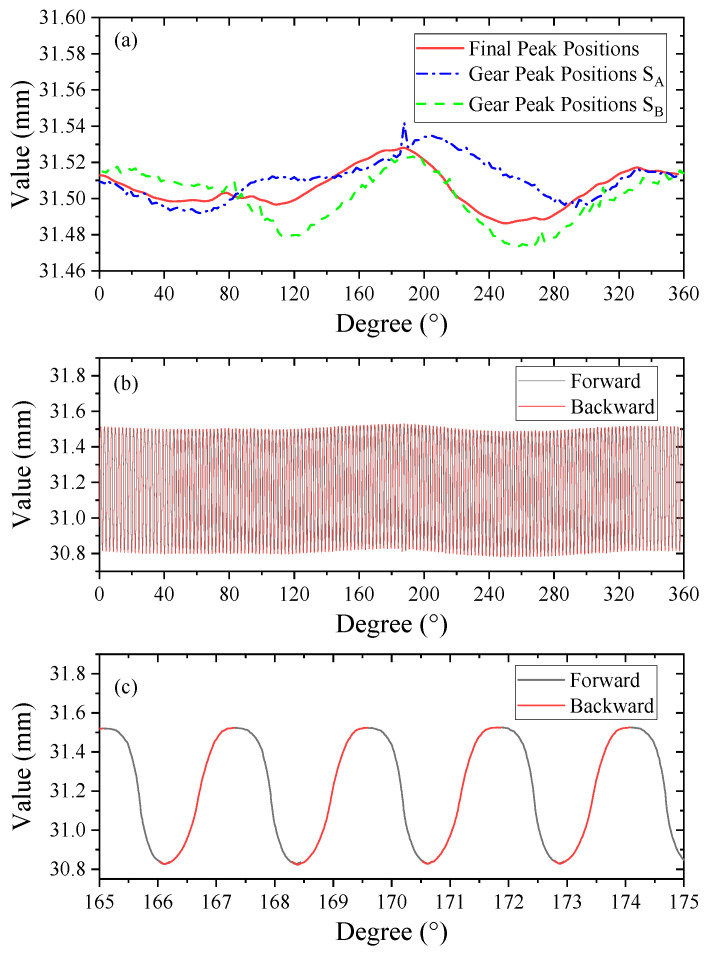
(**a**) Two-point method result; (**b**) the final flexspline profile; (**c**) enlarged profile.

In Figure 6b, the position of the tooth top does not completely coincide at both ends because the rotation error cannot be totally removed. In order to splice the tooth flank profiles on both sides accurately, it is necessary for the two curves to seek an accurate peak as their connecting point. The two-point method is used for the error separation with a chromatic confocal sensor (STIL, CCS PRIMA), whose resolution is about 2 nm. The chromatic confocal sensor is installed with an inclined angle of 179.56°. Finally, the gear peak positions are extracted after the error separation process, as shown in Figure 7a. Using the gear peak positions, the tooth flank profiles on both sides were spliced together to obtain the complete final flexspline profile in Figure 7b, whose enlarged curve is shown in Figure 7c. It can be seen that the tooth top coincides at both ends, showing better conference.

## 4. Results

**Figure 8:**
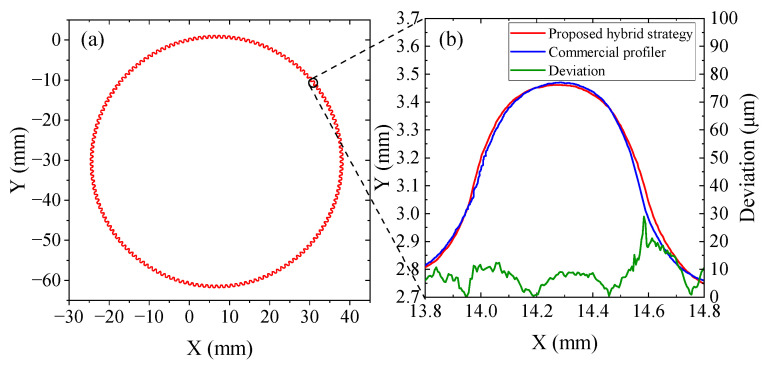
(**a**) Obtained global flexspline profile with 160 gear teeths; (**b**) local comparison between hybrid strategy and commercial profiler.

The final circular outline of the flexspline is shown in Figure 8a. The teeth periodically surround the base circle. The result shows that the average tooth height is 703 μm, the average pitch is about 1230 μm, the average outside diameter is 62,967 μm, and the modulus is 391 μm, which are consistent with the theoretical design of the flexspline. Indeed, the profile measuring accuracy mainly lies in the high accuracy of the triangulation laser sensor and the chromatic confocal displacement sensor.

Furthermore, in order to verify the correctness of the measured tooth profile, a commercial ultraprecise profiler SEF680 is used to measure the same flexspline. The measuring range of SEF680 is 50 mm × 100 mm with a measuring force of 30 mN. Its tip radius is about 25 μm, and the axial displacement measuring accuracy is better than 0.8 μm. In fact, it is also a three-coordinate method by scanning the XY axes. As the moving straightness accuracy is 1 μm/100 mm, the profile measuring accuracy reaches 1 μm for these workpieces, whose size is less than 100 mm. For the gear profiles, the profiler is also most used because of its high stability and accuracy.

The comparison between the proposed system and SEF680 is shown in Figure 8b. Two tooth profiles are highly consistent, and their deviation is less than 20 μm. Compared with SEF680, the profile obtained by the proposed system is smoother without obvious jitters. On the other hand, the profiler cannot move at a high speed of more than 0.1 mm/s to avoid collision between the measuring probe and the flexspline. Hence, the measurement efficiency is higher with the proposed system without collision risks. In conclusion, the measuring results show good accuracy, feasibility, and efficiency of the hybrid measuring strategy to obtain the gear profile with a small modulus. These advantages make the proposed strategy hopeful to replace some applications of the huge, expensive profiler.

## 5. Conclusions

This paper proposed a hybrid method for measuring the profile of micro gear structures with a contact probe and a noncontact optical sensor. The auxiliary lifting strategy and error separation method are used to avoid contact damage and eliminate measuring error. The two-point method is employed to eliminate the installation error and rotary error to extract accurate tooth peak positions. Additionally, the flexspline tooth profiles are connected with the tooth peak positions. By comparing the results with the SEF680 commercial profiler, the profile deviation is within 20 μm, which verifies the reliability of the measurement accuracy. In conclusion, this research provides a feasible method for meeting the accuracy and efficiency requirements of flexspline profile measurement and demonstrates its potential to be used for similar measurements of rotary components with complex micro surfaces.

## Figures and Tables

**Table 1:** Calculation of parameter values.

Parameter	e (mm)	α_0_ (°)	s (mm)	p (mm)
Forward	0.2896	1.476	1.102	−39.13
Reverse	0.2898	−1.388	1.140	−39.16
Average	0.2897	±1.432	1.121	−39.14

## Data Availability

Data will be made available upon request.
